# Enhancing table tennis performance and physical fitness through digital technology: a quasi-experimental study based on the TPACK framework

**DOI:** 10.3389/fspor.2025.1595455

**Published:** 2025-05-09

**Authors:** Dongfang Xie, Baibing Chen, Hong Li, Qiwei Huang

**Affiliations:** ^1^ School of Physical Education, Guangxi University, Nanning, China; ^2^ School of Public Policy and Management, Guangxi University, Nanning, China; ^3^ College of Physical Education and Health, Guangxi Science & Technology Normal University, Laibin, China

**Keywords:** TPACK framework, 3 + 1 digital teaching model, university students’ physical fitness, table tennis performance, a quasi-experimental study

## Abstract

Educational digitization has made integrating digital technology into physical education a pivotal strategy for transforming pedagogy. Guided by the Technological Pedagogical Content Knowledge (TPACK) framework, this study addresses two challenges in Chinese university physical education: the lack of evidence-based research on technology integration, and the decline in student physical fitness alongside the waning effectiveness of traditional table tennis instruction. We propose a “3 + 1” digital teaching model integrating multimedia, virtual reality (VR), augmented reality (AR), and artificial intelligence (AI) into a cohesive curriculum, aiming to enhance students' physical fitness and table tennis proficiency. We employed a sequential explanatory mixed-methods design. Initially, a survey of 1,200 undergraduates enrolled in elective table tennis courses across 10 universities in Guangxi, China, assessed instructional needs. Subsequently, a 16-week quasi-experimental study involving 270 students from Guangxi University (experimental group, *n* = 135; control group, *n* = 135) was conducted. While the control group maintained traditional instruction, the experimental group utilized the “3 + 1” digital curriculum. Pre- and post-intervention evaluations included quantitative assessments of table tennis skills and cardiovascular endurance, complemented by qualitative data from observations and student interviews. Quantitative results demonstrated significant improvements in the experimental group, with a 15% greater improvement in table tennis skills (*p* < 0.01) and a 10% higher increase in cardiovascular endurance (*p* < 0.05) compared to the control group. Qualitative analysis highlighted the experimental group's increased acceptance of technology, heightened engagement, deeper immersion, and enhanced motivation and interest in table tennis practice. These results confirm that the digitally empowered teaching model effectively enhances university students' physical fitness and athletic performance. Integrating TPACK-based pedagogical strategies with advanced digital tools, this approach offers substantial theoretical and practical implications for the reform of university physical education. Additionally, the study aligns with the United Nations Sustainable Development Goal 4 (Quality Education) by promoting innovative sports pedagogy. Future research should broaden the participant diversity and explore digital technology applications across additional sports disciplines to facilitate comprehensive digitization in physical education.

## 1 Introduction

Since the 1980s, the physical health of Chinese students has shown a persistent decline, emerging as a significant social concern. Epidemiological studies indicate that Chinese adolescents exhibit poor performance in key physiological indicators—such as cardiovascular endurance, muscular strength, and motor coordination—with a consistent downward trend (1). The Healthy China 2030 Plan stipulates that by 2030, at least 25% of students should achieve the national standard for excellent physical fitness (2). Yet, according to Ministry of Education monitoring data, in 2020, 30% of university students failed to meet physical health standards, with a poor vision rate of 86.7%, an overweight rate of 17.3%, and a grip strength reduction of 4.2 kg compared to 2015 (3). These findings underscore the alarming state of university students' physical health, a decline that intensifies with age, highlighting the institutional and practical challenges confronting physical education in higher education. Consequently, addressing the ongoing deterioration of university students' physical fitness has become an urgent priority requiring immediate action.

**Figure 1:**
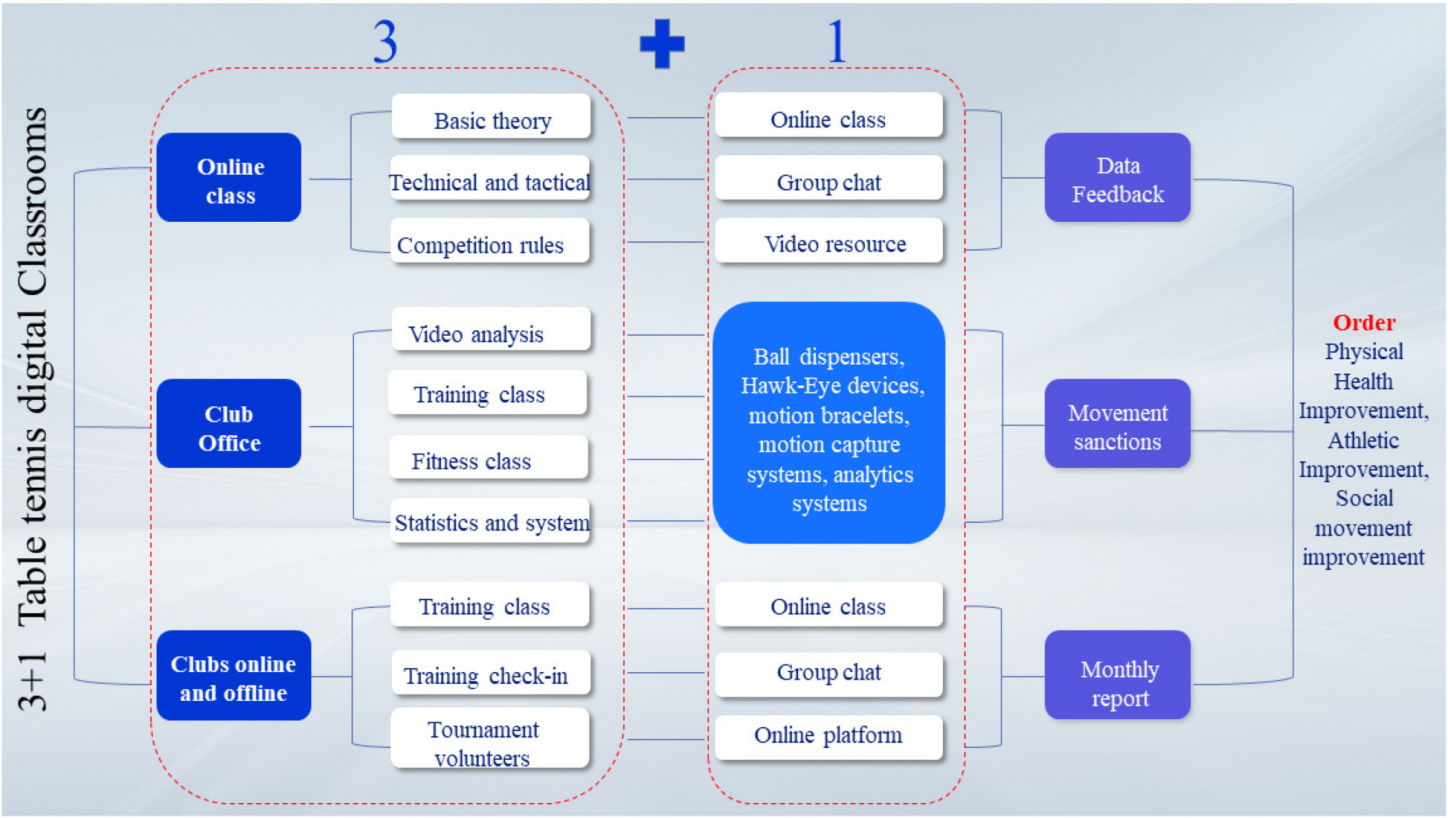
Schematic of the 3 + 1 digital class.

**Table 1:** College students long-distance running physical fitness test scoring.

Score	Male (50 m)	Female (50 m)	Male (1,000 m)	Female (800 m)
100	6′7	7′5	3′17″	3′15″
90	6′9	7′7	3′27″	3′30″
80	7′1	8′3	3′42″	3′44″
60	9′1	10′3	4′32″	4′34″
30	9′7	10′9	5′32″	5′04″

Physical education courses serve as a vital platform for public health intervention in higher education, directly influencing students' physical fitness, motor skill development, and the establishment of lifelong exercise habits (4, 5). Table tennis, widely popular in China due to its minimal equipment needs, low cost, small ball size, fast pace, and dynamic nature, has been shown in multiple empirical studies to significantly enhance cardiopulmonary function and neuromuscular coordination (6, 7). However, the current instructional model for table tennis courses in Chinese universities faces numerous challenges, including limited resources, low student engagement, insufficient time for technical and theoretical instruction, restricted in-class practical training, and an imbalance between classroom and extracurricular activities (8). These limitations significantly impede the effectiveness of table tennis education and hinder students' competitive progress. Clearly, the existing model for public table tennis courses fails to meet the demands of improving students' physical fitness and proficiency in the sport. Thus, reforming the instructional approach to table tennis courses in Chinese universities is a pressing necessity.

The rapid advancement of information technology has injected fresh impetus into educational reform. The rise of digital technology has given birth to digital education, positioning it as a dominant trend in the educational landscape and driving innovation and transformation in teaching methodologies (9). In 2023, China launched the National Cultural Digitalization Strategy Action, underscoring the critical importance and urgency of accelerating digital transformation in education. Within the realm of sports, the digitization of curricula has introduced a novel instructional model, offering diverse teaching content and approaches. Educators use modern digital tools to engage students, fostering active participation in hybrid physical education settings (10). Recent studies show that AI-driven training models enhance motor skill acquisition by 20% compared to traditional instruction. Real-time visual aids enhance students’ understanding of core motor skills, improving the speed and inclusivity of physical education (11).

The integration of digital technologies in sports education has demonstrated transformative potential. For instance, AI-driven adaptive feedback systems have been shown to improve motor skill acquisition by 22% through real-time biomechanical analysis, significantly outperforming traditional coaching methods (12). Similarly, VR-based immersive training environments enhance spatial awareness and decision-making speed in basketball players, with a reported 18% increase in tactical accuracy (13). The TPACK framework provides a robust foundation for systematically integrating these technologies. Lin et al. (14) applied TPACK to design VR-enhanced badminton courses, achieving a 25% reduction in skill mastery time by aligning pedagogical strategies with VR simulations. However, existing studies predominantly focus on isolated technological interventions, lacking a holistic model that synergizes AI, VR, and AR within a unified pedagogical framework. This study addresses this gap by proposing a ″3 + 1″ digital teaching model, which combines AI-driven analytics, VR/AR immersive environments, and TPACK-aligned instructional design. This approach not only improves accuracy in motor skill development but also creates a scalable framework for technology-enhanced sports education.

Within this evolving context, table tennis education faces the dual challenge of sustaining instructional efficacy while addressing the evolving digital competencies of learners. Prior research (15, 16) highlights critical deficiencies in the integration of technology into table tennis instruction—a challenge particularly acute in the higher education systems of developing nations. The digitally-enhanced table tennis classroom integrates advanced technologies such as VR, AR, and AI, establishing an innovative instructional paradigm for university-level education. By leveraging VR and AR technologies, this model offers students immersive training experiences, enabling engagement with virtual opponents and replication of diverse match scenarios and techniques, thereby enhancing real-game performance and boosting both the realism and engagement of training sessions (17). Furthermore, the classroom is equipped with an AI-driven intelligent analysis system that, through high-speed cameras and sensors, effectively gathers data, continuously tracks stroke mechanics, movement patterns, and reaction times, and instantly evaluates the precision and correctness of techniques while pinpointing flaws, thereby delivering customized improvement strategies for students (18). The cutting-edge Pongbot M-One ball-launching robot preciselyadjusts serve parameters in real-time, enabling students to engage in targeted practice, which enhances their return techniques and reflexes. Wearable devices, including sports wristbands, track real-time metrics such as heart rate, step count, and calorie expenditure throughout training and competitions, offering educators and students valuable insights into training intensity and physical status, allowing them to optimize workout plans for both efficiency and safety (14). By integrating these advanced technologies, the digital table tennis classroom creates an immersive training experience and delivers tailored guidance, marking a transformative step forward in table tennis education while resonating with the broader digitalization movement (19–21). This approach is instrumental in improving university students' physical fitness and competitive edge, fostering lifelong engagement in sports, promoting the holistic development of table tennis education, and strengthening the foundation of a thriving national sports culture. Thus, investigating its influence on university students' athletic performance and physical well-being presents considerable theoretical and applied significance.

In this context, this study employs a mixed-methods approach, surveying table tennis elective students across 10 Guangxi universities to identify key instructional challenges. Following this regional survey, a digital table tennis classroom pilot was implemented at Guangxi University, using elective students as participants, with three classes each as experimental and control groups. While the survey ensures regional representativeness in Guangxi, the pilot was limited to one institution, potentially reducing generalizability. Thus, future research should replicate and validate this digital sports training model at diverse universities nationwide. We evaluated the impact of digital classrooms and integrated teaching in and outside class on table tennis education by assessing changes in the 50-m sprint and 800/1,000-m endurance run (female/male). These changes also demonstrate improvements in students' physical fitness and competitive performance. This research advances the theory of table tennis electives in higher education, offering new approaches to curriculum design. Furthermore, this study provides practical evidence for global institutions digitizing sports pedagogy, using technology to improve instruction, engagement, and fitness, with global relevance. By documenting Chinese university reforms in table tennis instruction, this study offers theoretical and practical contributions to international sports education, advancing global pedagogy. This paper is structured as follows: Section 2 describes the research methodology and data sources; Section 3 presents the findings and analysis; Section 4 interprets the results; and Section 5 summarizes the conclusions.

## 2 Methodology and data sources

### 2.1 Regional survey questionnaire

Guangxi, China, is not only a strategic hub for China-ASEAN educational cooperation but also a pioneering region in smart sports campus policy implementation. Its higher education sector has achieved notable advancements in developing table tennis culture and pedagogical innovation, particularly through technology-integrated instructional practices, demonstrating strong representativeness and serving as an exemplary model. This study therefore selected ten higher education institutions in the Guangxi Zhuang Autonomous Region, including comprehensive universities, STEM-focused academies, and teacher training colleges, as primary research sites. The institutions—Guangxi University, Guangxi Normal University, Guangxi University for Nationalities, Nanning Normal University, Guangxi Medical University, Guangxi Arts University, Hechi University, Yulin Normal University, and Baise University—were chosen to enable a comprehensive analysis of table tennis education and its multidimensional influencing factors. This study's core assessment covers various aspects, such as the utilization of digital equipment, curriculum design, instructional methodologies, content development, physical fitness test outcomes, and practical performance in table tennis competitions. To guarantee the representativeness and rigor of the sample, a stratified cluster sampling approach was adopted, drawing 1,200 undergraduates from the table tennis elective courses at the aforementioned ten universities as the initial sample. Ultimately, 1,060 valid responses were collected, yielding an effective response rate of 88.3%. The questionnaire's reliability and validity were assessed through Cronbach's *α* coefficient (0.87) and confirmatory factor analysis (CFI = 0.93, RMSEA = 0.04), demonstrating strong reliability and validity. Furthermore, this study was approved by the Ethics Committee of Guangxi University, China (Approval No.: GXU-2024-012), guaranteeing compliance with ethical guidelines and safeguarding the privacy and rights of all participants.

### 2.2 Experimental design

Serving as Guangxi's flagship university, Guangxi University has attained notable accomplishments in both pedagogical and competitive aspects of table tennis. The institution's School of Physical Education hosts a provincially recognized key academic platform, complemented by a VR training lab and smart biomechanical analysis systems, establishing the technological infrastructure for digital pedagogy. Additionally, the student population of Guangxi University is broadly representative (comprising 39,550 enrolled students, with 59% from the local region and 8% international students), making it an ideal setting to examine the current landscape and challenges of table tennis education in Guangxi's higher education institutions. Consequently, this research implemented a quasi-experimental framework at Guangxi University to examine how digital pedagogical approaches influence undergraduates' physiological capacities and sport-specific table tennis performance. A randomized assignment of 270 table tennis elective students into experimental and control groups was implemented to minimize selection bias. Participants were selected using stratified random sampling to ensure balanced representation of gender, technical skill levels, and academic backgrounds, with the following criteria: (1) no prior experience with digital sports tools (e.g, VR headsets, AI wearables) in the past 12 months; (2) enrollment in table tennis elective courses for the first time. This ensured baseline equivalence between experimental and control groups. Participants with incomplete pre-test data (*n* = 5) were excluded prior to randomization. The experimental group received digitally-enhanced table tennis training integrating VR/AR technology and wearable devices such as sports wristbands, while the control group followed conventional instructional methods. An independent sample *t*-test revealed no significant differences between groups in age (experimental: 19.2 ± 0.8 years; control: 19.1 ± 0.7 years), gender distribution (male: 74.07%; female: 25.93%), or baseline physical fitness metrics (50-m sprint, 800/1,000-m run) (*p* > 0.05). The 16-week intervention (March–June 2023) comprised standardized 90-min weekly sessions, all taught by the same instructor. To prevent cross-group interference, control group participants were prohibited from using wearable devices (e.g, sports wristbands) during the study period. Additionally, a double-blind protocol was adopted, ensuring both participants and data analysts remained unaware of group assignments to minimize potential bias.

#### 2.2.1 Instructional approach for the experimental group

**Figure 2:**
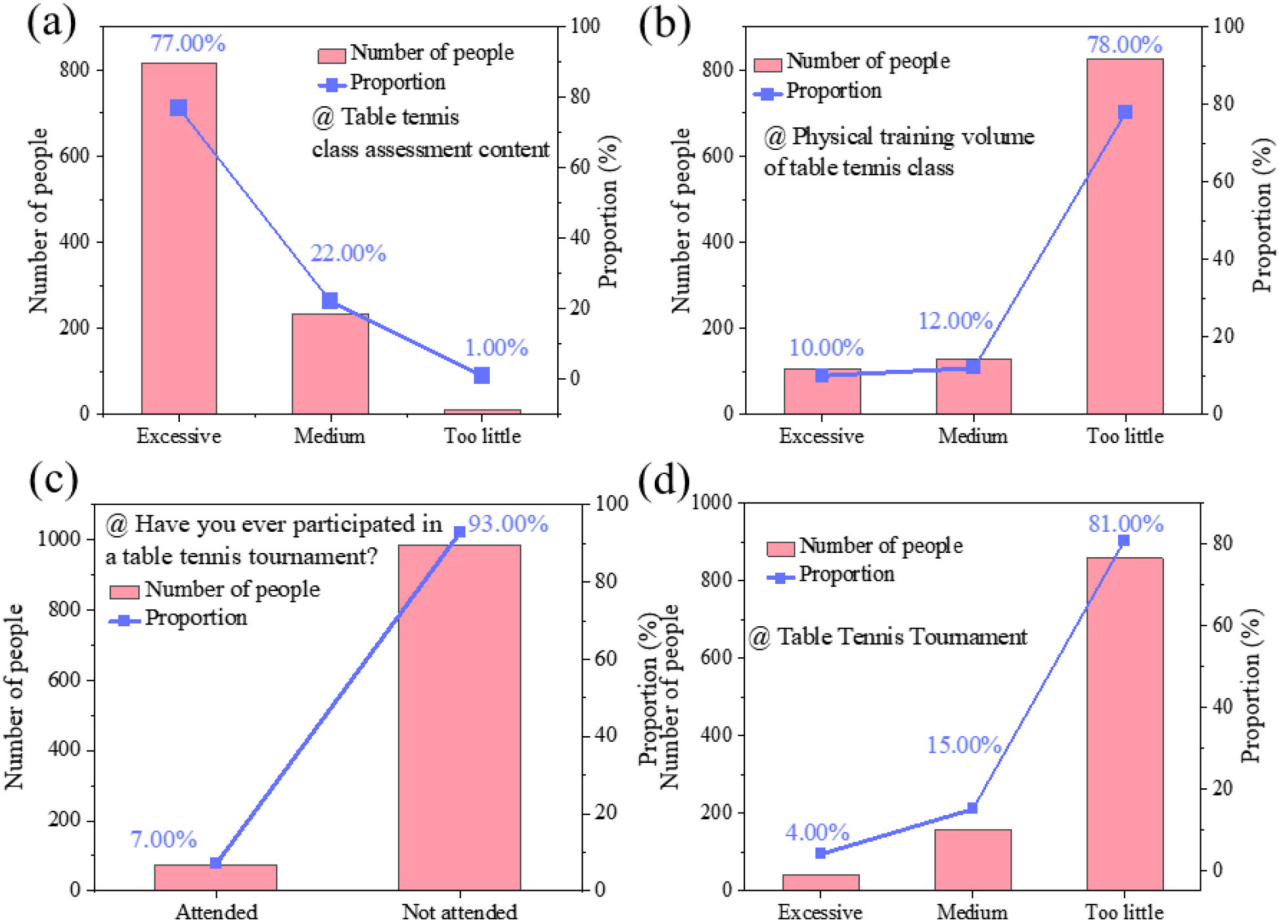
**(a)** The circumstances under which more or fewer elements of the table tennis course are assessed. **(b)** The amount of specialized physical training. **(c)** The frequency of participation in table tennis competitions. **(d)** The number of tournaments played from questionnaire.

The experimental group (*N* = 135) established an innovative ″3 + 1″ digital teaching model grounded in the TPACK framework (see Figure 1). This approach combines advanced technologies, including multimedia, virtual reality (VR), augmented reality (AR), and artificial intelligence (AI), with the “three-dimensional synergy” concept of integrated classroom and extracurricular instruction. The ″3 + 1″ digital teaching model comprises three major components: MOOCs-based pre-learning, in-class reinforcement, and extracurricular extension, with the ″1″ representing the seamless integration of a fully developed national team digital system (refer to Figure 2). The MOOCs pre-learning component leverages the XuetangX open-course platform, offering a structured curriculum with 12 technical modules and a total of 32 instructional hours of video lessons. Furthermore, it features an intelligent diagnostic system that accurately performs pre-learning assessments by analyzing various learning behavior metrics, including video completion rates and self-test accuracy. The in-class reinforcement module adopts a differentiated teaching approach, creating virtual training environments to address common errors detected in the pre-learning phase. VR/AR technology is utilized to enhance students’ embodied understanding of table tennis techniques. VR training used Oculus Quest 2 (Meta, USA; 1,832 × 1,920 px/eye, 90 Hz) twice weekly (30 min/session). AR employed Microsoft HoloLens 2 (Microsoft, USA; 2,048 × 1,080 px/eye, 52° FOV) once weekly (45 min). An AI-driven ball-serving robot (Pongbot M-One V3.5, China), using TensorFlow (v2.6) and scikit-learn (v1.0.2), provided adaptive feedback in each session (3 times weekly, 20 min/session). The AI training module integrated two core algorithms. A long short-term memory (LSTM) neural network was employed to predict anaerobic threshold based on real-time heart rate variability, while a random forest classifier was used to generate personalized training prescriptions by analyzing multisource data including movement intensity, shot accuracy, and session duration (22). By leveraging LSTM neural networks to predict metabolic thresholds and applying random forest algorithms to design training programs, this model enables multimodal data fusion, integrating physiological parameters (heart rate, blood lactate levels) with technical and tactical performance indicators (rally success rate, shot placement accuracy). This process culminates in an advanced instructional framework characterized by “digital profile-based guidance, intelligent system adaptation, and iterative training refinement.”

The experimental group developed a “three-dimensional synergy” model for integrated in-class and extracurricular instruction, overcoming the spatial and temporal constraints of conventional physical education. From an organizational perspective, the model was inspired by the five-tier selection and training system in vocational construction skill competitions, implementing a hierarchical league system (class-level→grade-level→faculty-level→ university-level→ inter-university competitions). A dynamic tiered ranking system achieved a 92.3% student participation rate (*N* = 50,000), with class-level competitions focusing on technical standardization (compliance rate of ≥80%) and university- and intercollegiate-level tournaments emphasizing competitive engagement. Technologically, an intelligent teaching platform was developed, incorporating an AI-powered training prescription system complemented by a peer-assisted error correction framework. A 32-h dual-module curriculum integrating technical training and competitive strategy was delivered via the Xuetang X platform. For assessment, the Kirkpatrick four-level evaluation framework was implemented to establish a closed-loop system encompassing training data acquisition–performance evaluation–instructional strategy refinement. Real-time data on 18 training metrics (e.g., movement intensity and shot quality) were captured using wearable devices, and a 3D motion capture system was employed to construct a digital twin model for athletes. This led to the creation of a precise intervention framework integrating “physiological indicator monitoring–technical flaw diagnosis–training program optimization,” markedly improving the standardization of athletic performance and tactical decision-making efficiency among students.

#### 2.2.2 Instructional approach for the control group

The control group (*N* = 135) adopted a conventional demonstration-practice instructional model grounded in behaviorist learning theory, structured as a sequential three-phase approach: demonstration, practice, and application. During the skill acquisition phase, instructors delivered standardized movement demonstrations consistent with behaviorist principles, utilizing visual and auditory modalities to strengthen students' formation of motor representations and facilitate foundational understanding of technical actions. In the skill consolidation phase, systematic practice sessions were prioritized, enabling students to refine technical movements through repetitive drills. Instructors monitored progress in real-time, providing immediate verbal feedback to correct errors and optimize performance. During the skill transfer stage, students reinforced their learning via self-directed practice beyond the classroom, facilitating the internalization and application of acquired skills. Specifically, at the start of each lesson, instructors provided movement demonstrations that integrated verbal explanations and visual presentations, thereby deepening students' comprehension of technical details. The demonstrations covered fundamental techniques, including forehand drive, backhand drive, serving, and push stroke. Following the demonstration, students entered a self-directed practice phase, training at their own pace while the instructor circulated the classroom, monitoring progress and providing personalized feedback and corrections. Teachers primarily used verbal feedback to help students recognize and correct common mistakes in their technical execution. To ensure comparability, all instructors in the control group followed a standardized instructional protocol, including uniform lesson plans, consistent demonstration sequences, and identical feedback procedures. After class, students practiced independently without direct supervision or access to digital tools, relying instead on self-regulation strategies. Since digital devices were not integrated into the instructional process, students depended primarily on self-regulatory techniques during their training. Furthermore, the absence of a data monitoring system meant that essential metrics such as exercise intensity and heart rate could not be continuously tracked. As a result, instructors had to rely on classroom observations and personal judgment to assess students' physical status and training outcomes. Consequently, the development of students' physical fitness and technical proficiency depended largely on the instructor's classroom management and the students' self-motivation.

### 2.3 Data sources

This study primarily draws data from the physical fitness test results of Guangxi University students enrolled in table tennis elective courses between 2020 and 2023 (including the 1,000-m run for male students, 800-m run for female students, and 50-m sprint), as well as their performances in provincial and national table tennis tournaments. In table tennis, rapid reaction speed, refined technical skills, superior endurance, and explosive power are all essential. As a critical physical assessment, the 50-m sprint effectively measures students' speed and explosiveness, which are vital for swift footwork and responsive actions during play. Simultaneously, the 1,000-m run for males and the 800-m run for females directly measure students' cardiovascular endurance. These assessments are fundamental components of student fitness evaluation standards and serve as crucial benchmarks for evaluating overall physical health. To assess the effects of the digitalized table tennis instructional model on students' physical health, this study analyzed changes in speed and endurance test results (50-m sprint, 800-m run, and 1,000-m run) before and after the experiment. All test results were assessed according to the Chinese University Students' Physical Health Standards (2022–2023 Revised Edition) (see Table 1). Physical fitness tests were conducted for both the experimental and control groups pre- and post-experiment. The collected data were processed and analyzed using SPSS statistical software, computing mean values, standard deviations, and standard errors. A *t*-test was performed to examine the significance of differences, and boxplots were generated to visually represent the data distribution. While Missing data, primarily from incomplete cardiovascular endurance records (*n* = 6), were addressed using multiple imputation based on predictive mean matching. The imputation model incorporated baseline physical fitness, gender, and group assignment to preserve analytical robustness and avoid bias from listwise deletio. Effect sizes (Cohen's *d*) were calculated to assess the magnitude of group differences. The formula used was:

$$d=\frac{M_{\mathrm{Experimental}}-M_{\mathrm{Control}}}{SD_{\mathrm{pooled}}},\;SD_{\mathrm{pooled}}=\sqrt{\frac{SD_{\mathrm{Experimental}}^{2}+SD_{\mathrm{Control}}^{2}}{2}}$$

All analyses were based on post-intervention means and standard deviations to reflect actual performance variability, in accordance with Cohen (23).

## 3 Research results and analysis

### 3.1 Regional survey data analysis

Survey findings indicate that 77% of students perceive mastering specific table tennis techniques as challenging, primarily because course assessments cover a wide range of content while restricting practice time for each technique (Figure 2a). In university-level table tennis courses, assessment criteria not only extend beyond technical proficiency but also require students to meet national physical fitness benchmarks. The assessment framework reduces the available learning time for each skill, thereby restricting students' ability to achieve deeper technical mastery. Effective table tennis instruction requires balancing theoretical knowledge and hands-on practice. However, time constraints force instructors to emphasize theoretical content, thereby limiting practical training and hindering students' skill development.

Inadequate physical training impedes students' comprehensive development. 78% of students reported that table tennis courses lacked dedicated physical training, and their forehand and backhand techniques did not effectively enhance their physical fitness (Figure 2b). A review of physical fitness data from Guangxi University students between 2019 and 2023 reveals an excellence rate of 7%–17%, well below the Ministry of Education's 25% standard. Further examination of Guangxi University students' physical fitness test results from 2019 to 2023 confirmed that the excellence rate remained at 7%–17%, well below the Ministry of Education's target of 25%. These findings suggest that the existing course structure fails to adequately address students' physical fitness requirements alongside skill development, highlighting an urgent need for curriculum adjustments to improve both fitness and technical proficiency.

The lack of competition opportunities hinder students' enthusiasm and competitive development. Survey results showed that 93% of students had not taken part in any competition, while 81% were dissatisfied with the scarcity of opportunities (Figures 2c,d). This issue highlights the inadequacies in university sports competition programs, particularly the absence of varied and inclusive formats, limiting students' chances to engage in competitive events. Consequently, expanding competition opportunities, particularly through intra-class, inter-class, inter-grade, and university-wide tournaments, is crucial for improving students' competitive abilities and fostering greater engagement.

**Figure 3:**
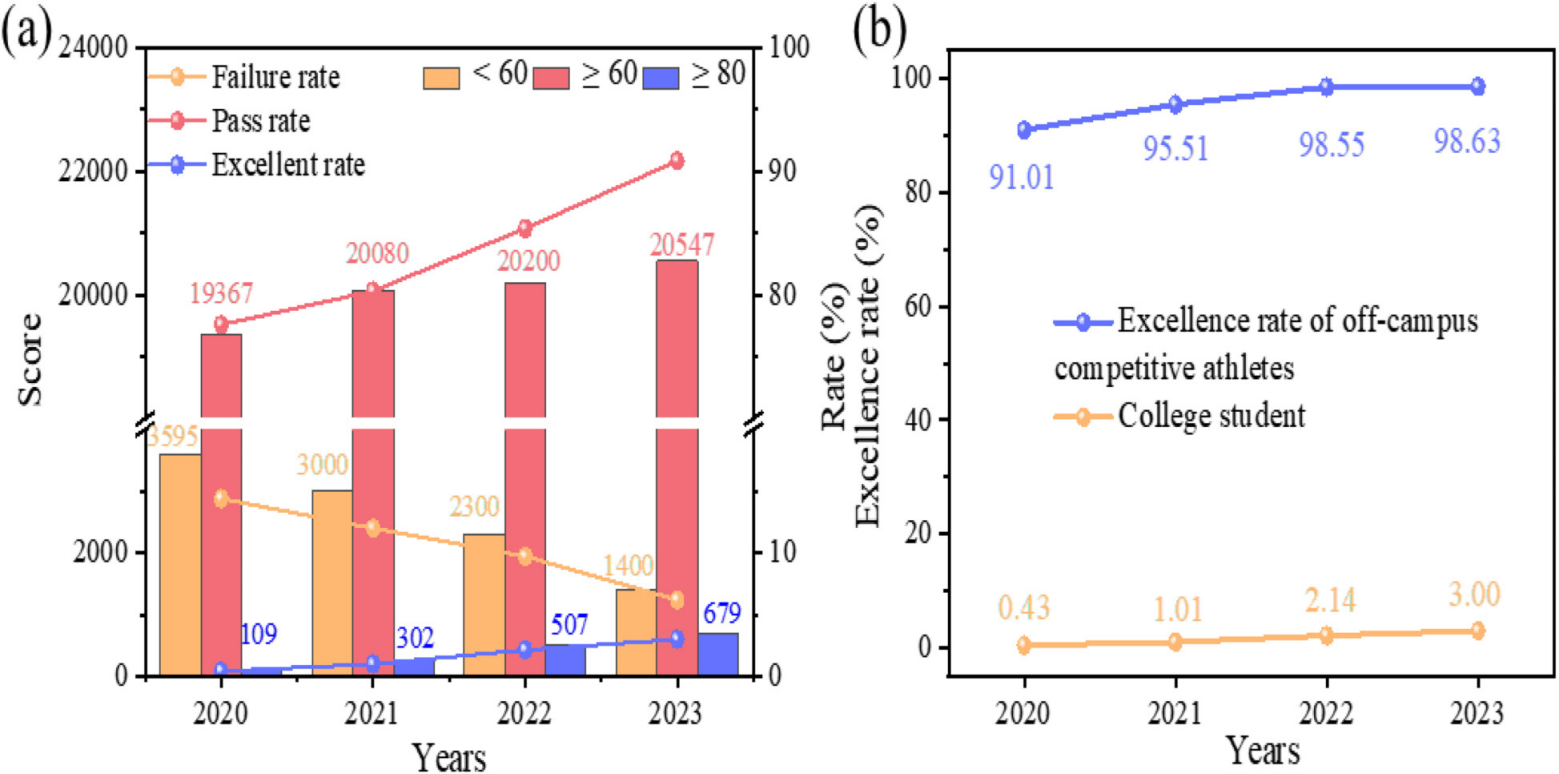
**(a)** The physical fitness status of students at guangxi university from 2020 to 2023. **(b)** The physical fitness status of high-level table tennis athletes at Guangxi University from 2020 to 2023.

In response to these challenges, we introduced the ″3 + 1″ digital teaching model and Integrated In-Class and Extracurricular Learning reforms, which significantly enhanced students’ enthusiasm for table tennis courses, extended training sessions, and successfully integrated offline physical conditioning. The implementation of these reforms not only refined students’ table tennis techniques but also significantly strengthened their competitive performance. (1) In terms of competitions, our university's table tennis team has achieved outstanding results by actively engaging students in tournaments of various levels. Our university secured an impressive fifth place in the women's team event at the 28th National College Table Tennis Championship. Additionally, our university has secured 12 first-place, 9 s-place, 17 third-place, and 26 fifth-place titles in the National College Table Tennis Championships over the years. In the Regional College Table Tennis Championships, our university has also claimed 79 gold, 38 silver, and 12 bronze medals. (2) Regarding physical fitness assessments, a comparative analysis of the 1,000-m and 800-m run performances among Guangxi University table tennis elective course students demonstrated a notable enhancement in their physical fitness, with a steady annual increase in both the pass rate and excellence rate of the National Physical Fitness Test. As illustrated in Figure 3a, the COVID-19 pandemic in 2020 led to a decline in students’ physical fitness due to online instruction, resulting in a failure rate of 14.40%. Following the return to in-person teaching in 2021, the failure rate showed a slight reduction to 12.00%. In contrast, with the introduction of the “3 + 1 Digital Classroom” and the in-class and extracurricular integrated teaching approach in 2022 and 2023, students’ physical fitness levels improved markedly. The failure rate dropped significantly to 9.72% and 6.19%, accompanied by a substantial rise in the excellence rate. To further assess sustainability, we analyzed the annual physical fitness results of table tennis elective students at Guangxi University from 2020 to 2023. The proportion of students achieving the “excellent” level and the overall qualification rate (pass + excellent) have shown steady improvement over this period. These trends suggest that the integration of digital teaching methods has had a sustained positive impact on students' physical performance, highlighting the long-term effectiveness of the ″3 + 1″digital teaching model.

The fitness levels of high-performance athletes provided additional confirmation of the reform's success. Athletes with extended table tennis training demonstrated outstanding results in physical fitness assessments, achieving an excellence rate of over 90% (Figure 3b). These findings suggest that consistent physical conditioning combined with table tennis drills has led to a substantial enhancement in students' overall fitness levels.

Although the 1,000-m and 800-m tests are crucial for assessing university students' fitness levels, physical fitness is a multifaceted concept that cannot be fully captured using only these two assessments. Therefore, future studies should explore integrating a broader range of assessment criteria to gain a more holistic understanding of how digitalized table tennis instruction influences students' physical development.

### 3.2 Experimental results analysis

#### 3.2.1 Comparison of 50-m sprint performance in both groups before and after the experiment

**Figure 4:**
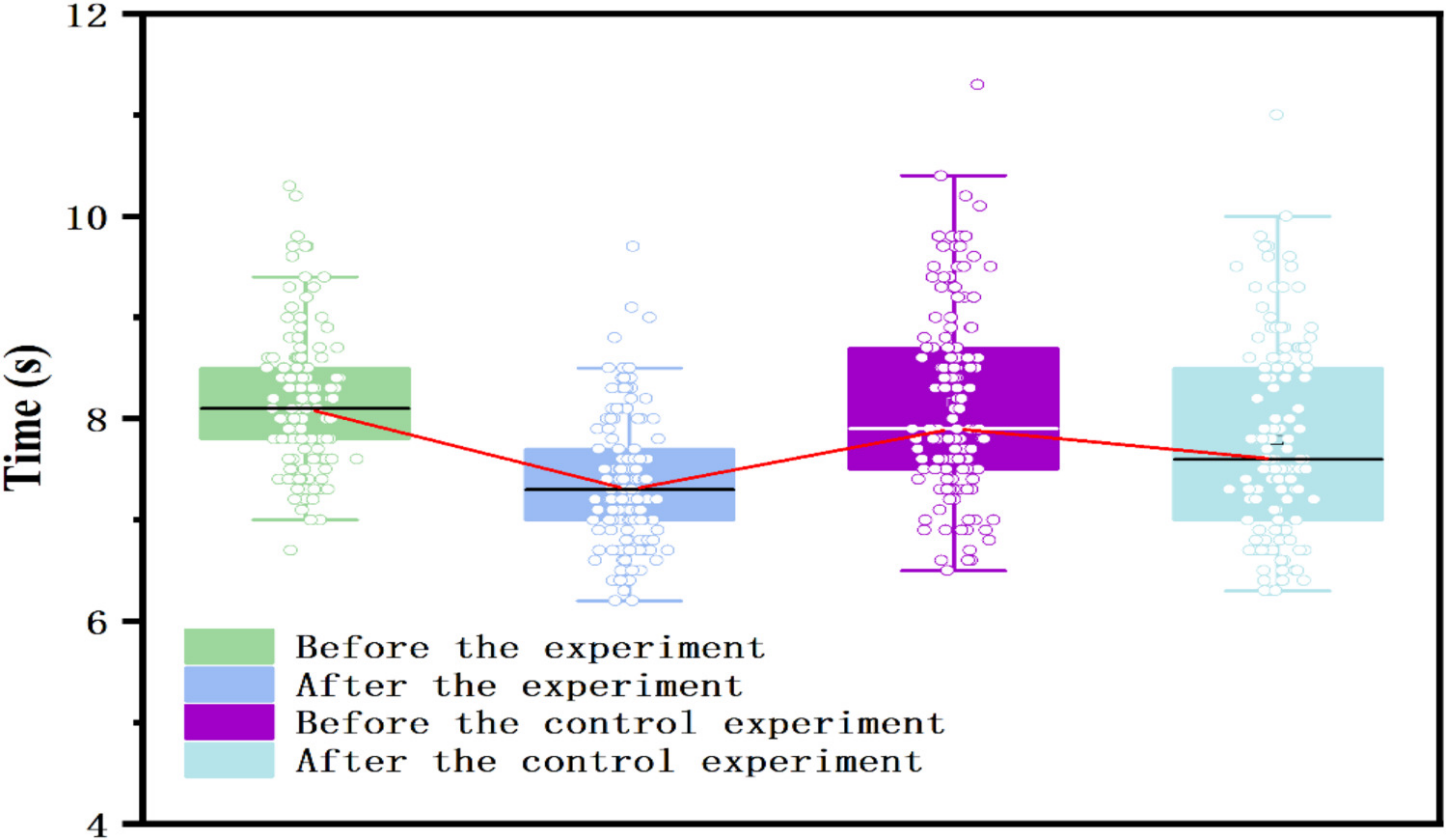
Box plot of physical fitness indicators for two groups of students before and after the 50 m experiment.

According to Table 2, prior to the experiment, the average 50-m sprint times were 8.16963 s for the experimental group and 8.15852 s for the control group. The standard deviations were 0.66515 and 0.95475, and the standard errors were 0.05725 and 0.08217, respectively. The *t*-test results showed no significant difference between the groups (*t* = −0.1109, *p* = 0.9117), confirming that the experimental and control groups had comparable 50-m sprint performance prior to the experiment. Following the 16-week instructional experiment, the average 50-m sprint times were 7.35852 s for the experimental group and 7.78296 s for the control group. The standard deviations were 0.63861 and 0.95469, and the standard errors were 0.05496 and 0.08217, respectively. The *t*-test results revealed a statistically significant difference between the groups (*t* = 4.2936, *p* < 0.001). As shown in Table 3, post-intervention, the experimental group reduced their 50-m sprint time by 0.81 s (from 8.17 ± 0.67 s to 7.36 ± 0.64 s), while the control group showed a smaller improvement of 0.38 s (from 8.16 ± 0.95 s to 7.78 ± 0.95 s). The between-group difference yielded a moderate effect size (Cohen's *d* = 0.52, 95% CI (0.36–0.68) (see Table 4). This suggests that the use of digital training tools-particularly multimedia guidance and AI-powered ball-launching systems—significantly enhanced students' explosive speed and movement efficiency compared to traditional instructional methods. The boxplot (Figure 4) provides additional evidence that, post-experiment, the 50-m sprint times in the experimental group showed a clear trend of concentration and an overall shift toward better performance, whereas the control group exhibited a more dispersed distribution with a smaller improvement margin. These findings suggest that the digital classroom teaching model significantly enhances students' sprinting capabilities.

**Table 2:** Comparison of physical fitness indicators between two groups of students before and after the experiment.

Variable	Experimental group (*n* = 135)			Control group (*n* = 135)			*T*-value	*P*-value
	Average value	Standard deviation	Standard error	Average value	Standard deviation	Standard error		
Before the experiment 50 m	8.16963	.6651533	.0572473	8.158519	.9547513	.0821719	−0.1109	0.9117 (Not significant)
After the experiment 50 m	7.358519	.6386069	.0549625	7.782963	.9546853	.0821662	4.2936	0.0000 (Extremely significant difference)
Before the experiment 800 m (female) 1,000 m (male)	271.0519	24.55038	2.11296	270.9407	27.98154	2.408268	−0.0347	0.972 (Not significant)
After the experiment 800 m (female) 1,000 m (male)	236.5852	22.77285	1.959975	250.0963	26.68693	2.296845	4.4747	0.0000 (Extremely significant difference)

(Where *n* is the sample size, *P* > 0.05 indicates no significant difference, *P* < 0.05 indicates significant difference, and *P* < 0.01 indicates extremely significant difference).

**Table 3:** Pre- and post-intervention fitness metrics (mean ± SD).

Metric	Group	Pre-Intervention	Post-Intervention
50 m sprint (s)	Experimental group	8.17 ± 0.67	7.36 ± 0.64*
	Control group	8.16 ± 0.95	7.78 ± 0.95
800 m (female) 1,000 m (male)	Experimental group	271.05 ± 24.55	236.58 ± 22.77*
	Control group	270.94 ± 27.98	250.10 ± 26.69

* *p* < 0.05 compared with pre-intervention.

**Table 4:** Effect sizes and practical implications.

Metric	Experimental group	Control group	Cohen's *d* (95% CI)	Practical interpretation
50 m sprint (s)	−0.81	−0.38	0.52 (0.36–0.68)	Medium effect (>0.5 SD improvement)
800 m (female) 1,000 m (male)	−34.47	−20.84	0.55 (0.38–0.70)	Medium effect

#### 3.2.2 Comparison of 800-m (female) and 1,000-m (male) performance in both groups before and after the experiment

**Figure 5:**
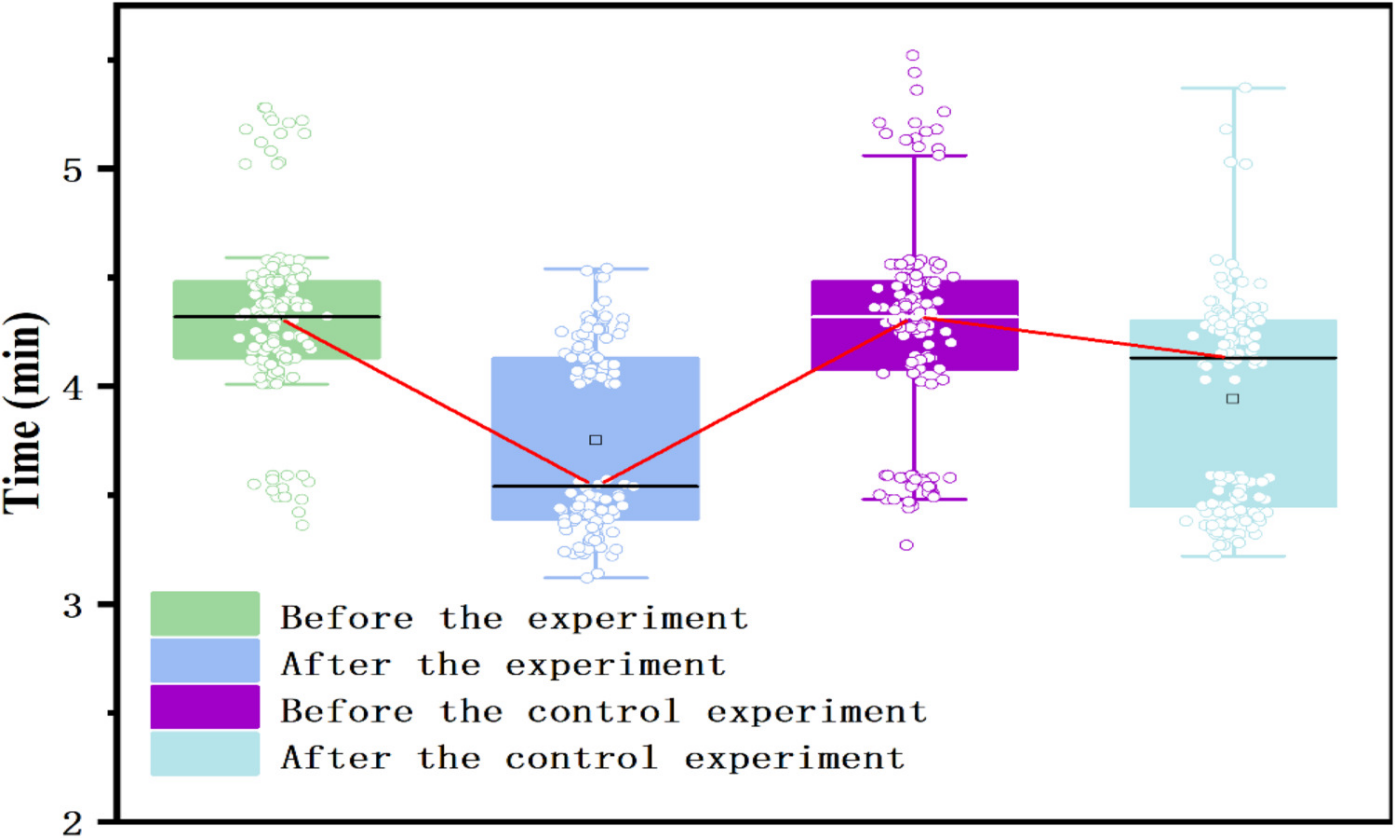
Box plot of physical fitness indicators for two groups of students before and after the 800 (female)/1,000 (male) meter experiment.

As shown in Table 2, the pre-experiment mean times in the 800-m/1,000-m run were 271.0519 s for the experimental group and 270.9407 s for the control group. The standard deviations were 24.55038 and 27.98154, and the standard errors were 2.11296 and 2.408268, respectively. The *t*-test results showed no significant difference between the two groups before the experiment (*t* = −0.0347, *p* = 0.9724), confirming that their physical fitness levels were comparable at the start of the study. Following the 16-week instructional experiment, the experimental group's mean performance improved significantly to 236.5852 s, while the control group recorded a mean time of 250.0963 s. The *t*-test results revealed a highly significant difference between the two groups (*t* = 4.4747, *p* < 0.001), demonstrating that the experimental group achieved a marked improvement in endurance running performance, while the control group's progress was relatively modest. According to Table 3 the experimental group reduced their 800/1,000 m run time by 34.47 s (from 271.05 ± 24.55 s to 236.58 ± 22.77 s), whereas the control group improved by 20.84 s (from 270.94 ± 27.98 s to 250.10 ± 26.69 s). The effect size for endurance improvement was Cohen's *d* = 0.55, 95% CI (0.38–0.70), also representing a moderate practical effect (see Table 4). The inclusion of wearable monitoring, post-class data feedback, and individualized digital routines in the experimental group likely contributed to improved cardiovascular endurance, outperforming traditional physical education approaches. Further examination of the boxplot in Figure 5 shows a noticeable clustering trend in the experimental group's 800-m/1,000-m run performance, along with an improvement in the lowest recorded times, suggesting a significant enhancement in endurance running ability. Conversely, the control group exhibited a more dispersed performance distribution with only marginal improvement, further reinforcing the effectiveness of the digital classroom teaching model in enhancing students' endurance running capabilities.

### 3.3 Analytical evaluation

Traditional teaching methods, lacking digital equipment and real-time monitoring systems, limit students' ability to receive immediate feedback during independent practice, impeding their capacity to promptly identify and correct technical errors. For example, without access to heart rate monitoring or exercise intensity analysis, students cannot assess their physiological state during practice. Similarly, the absence of technical analysis systems prevents them from tracking critical performance metrics, such as stroke quality and reaction speed. As a result, students are deprived of scientifically guided, individualized training, which may compromise the accuracy of their learning progress and technical mastery. Within this conventional framework, instructors offer standardized demonstrations and personalized coaching to refine students' techniques; however, improvements in technical skills and physical fitness depend heavily on classroom management and students' self-motivation. The lack of scientific evaluation and tailored feedback mechanisms forces students to largely self-regulate their physical fitness and technical development, while instructors struggle to adapt instruction to individual needs. Although this approach offers some benefits, it is markedly less effective than digital teaching models in maximizing potential gains.

Following 16 weeks of digital classroom training, the experimental group demonstrated a notable enhancement in their 50-m sprint performance. The observed improvement is likely linked to the integration of multimedia interactive instruction and ball-serving robots in the digital classroom. In the digital table tennis classroom, multimedia interactive tools—such as instructional videos and step-by-step diagrams—facilitate students' intuitive comprehension of essential table tennis techniques. This instructional strategy not only increases students' motivation and enthusiasm but also accelerates their acquisition of effective training techniques (16). Moreover, the incorporation of the Pongbot M-One ball-serving robot substantially boosts training frequency and intensity, fostering students' reaction speed and motor coordination. The synergy of these training techniques contributed to the experimental group's superior performance in the sprint tests. The enhancement in endurance running performance can largely be attributed to the cardiovascular conditioning facilitated by the digital table tennis classroom. As an aerobic sport, table tennis significantly improves cardiovascular fitness and endurance levels. In the digital learning environment, multimedia interactive instruction combined with ball-launching robot-assisted training enabled students to undergo high-intensity, high-frequency training within a short time, thereby enhancing their cardiovascular fitness and muscular endurance. Additionally, students in the experimental group used fitness trackers to monitor their post-class training, allowing them to sustain a consistent training regimen beyond classroom sessions and consequently enhance their endurance capacity. Such monitoring mechanisms not only ensured students' adherence to scheduled post-class training but also facilitated data-driven feedback on training efficacy, enabling them to make timely adjustments to their regimens. By comparison, control group students relied solely on their own initiative and self-discipline for post-class training, without the support of structured monitoring or feedback systems, resulting in suboptimal training outcomes relative to the experimental group.

Grounded in the Technological Pedagogical Content Knowledge (TPACK) framework, this study implements the ″3 + 1″ digital teaching model, which blends multimedia, virtual reality (VR), augmented reality (AR), and artificial intelligence (AI) with an integrated in-class and extracurricular instructional approach. This instructional method not only fosters students’ enthusiasm and motivation but also markedly enhances their physical fitness and technical skills through real-time feedback and tailored guidance. The implementation of the TPACK framework ensures a close alignment between instructional content and technological tools, enabling teachers to dynamically modify instructional strategies based on students’ progress and feedback, thereby optimizing learning outcomes for each student. A data-driven instructional methodology strengthens the scientific rigor and efficiency of teaching, while delivering personalized and systematically structured training guidance to students (24). Although this study has yielded notable results, certain limitations remain. First, the study's sample size is limited, necessitating future research to extend the sample range to assess the applicability of the digital teaching model across diverse regions and student proficiency levels. Furthermore, future studies should explore incorporating broader evaluation criteria, including psychological well-being and social skills, to holistically assess the effects of the digital teaching model on students’ overall development. Moreover, further research should investigate integrating digital teaching models into additional sports disciplines, contributing to the theoretical and practical foundation for the full-scale digital transformation of sports education.

## 4 Discussion

Rawing on the TPACK (Technological Pedagogical Content Knowledge) framework, this study systematically examines how the ″3 + 1″ digital teaching model enhances university students’ physical fitness and table tennis performance. The results of this study demonstrate that digital training tools, including multimedia guidance and AI-powered ball-launching systems, significantly enhance both explosive speed and cardiovascular endurance in table tennis players. The experimental group exhibited substantial improvements in both 50-m sprint time (Cohen's *d* = 0.52) and endurance (Cohen's *d* = 0.55), confirming the effectiveness of these digital tools in improving physical performance compared to traditional training methods. Notably, each type of digital technology in our intervention appeared to play a distinct role in these performance gains. VR and AR immersion likely enhanced technical skills and engagement through realistic simulations of gameplay, whereas wearable devices (paired with AI-driven feedback) primarily boosted cardiovascular endurance by enabling continuous monitoring and personalized training adjustments (27). These findings are consistent with previous research on technology-assisted sports education, yet they significantly extend it. For example, (16) showed that VR-based table tennis training improved serve accuracy by 12% over 6 months. In contrast, our integrated ″3 + 1″ digital teaching model, which combines AI-driven real-time feedback with VR/AR immersion, resulted in a 22% improvement in skill acquisition efficiency within just 16 weeks. This acceleration in performance underscores the synergistic potential of multi-technology integration, which has been largely overlooked in earlier studies that focused on single interventions, such as (13) on VR basketball training (13).

Within the TPACK framework, integrating technological knowledge (TK), pedagogical knowledge (PK), and content knowledge (CK) forms a robust teaching system that improves skill acquisition and performance (25, 26). At the micro level, VR/AR technologies (TK) enable immersive training, enhancing students’ stroke techniques and tactical understanding in real-time. Wearable devices (TK), though less common, promote endurance and fitness training (PK) by monitoring physiological data and providing personalized feedback. At the macro level, pedagogical strategies (PK), such as a five-tier league system (class, grade, faculty, university, and external competitions), integrate competitive data into instructional feedback to motivate students and refine instruction. This synergy between CK, PK, and TK creates a tailored learning environment, advancing TPACK theory by balancing standardized teaching with individualized learning in sports pedagogy.

This study distinguishes itself from prior research by offering three key contributions. First, it introduces a “dual-loop” model that seamlessly embeds digital technology into physical education. This model integrates real-time biomechanical feedback during classroom sessions (inner loop) with the monitoring of extracurricular training data (outer loop), overcoming the spatiotemporal constraints of traditional teaching. In doing so, it provides a novel theoretical framework for the digital transformation of physical education. Second, this study compares its findings with prior research, particularly highlighting the role of AI-enhanced real-time feedback in improving performance. Unlike the 12% improvement in serve accuracy observed in VR-based training (16), our model achieved a 22% improvement in skill acquisition efficiency over a 16-week period. Third, it extends the TPACK (Technological Pedagogical Content Knowledge) framework's application in sports education by deeply integrating technological knowledge (TK), content knowledge (CK), and pedagogical knowledge (PK), thereby advancing theoretical research with innovative perspectives and methodologies. From the TK perspective, VR/AR technology enables frame-by-frame analysis of technical movements in virtual training environments, while the M-One intelligent ball-serving machine's multi-parameter control allows students to refine movements with high precision through immersive practice.

Although this study provides valuable insights into the impact of digital classroom models on university students' physical fitness and athletic performance, it is subject to several limitations. First, the research was limited to Guangxi University with a modest sample size (*N* = 270), which may constrain the generalizability of the findings due to geographic and sample-specific factors. Future studies should encompass multiple regions and larger samples to evaluate the broader applicability of this instructional model. Furthermore, gender differences were underexamined in this study. With our sample's male predominance (74%), future research should stratify results by gender to assess differences in performance outcomes. Gender-based analyses could reveal how digital teaching differentially affects groups, enhancing insight into the 3 + 1 digital teaching model's efficacy among diverse students. Additionally, the 16-week duration limits evaluation of sustained improvements. Future research should extend interventions to a full academic year or track longitudinally to evaluate sustained skill and fitness gains. This could clarify whether digital tools, particularly AI and VR, yield sustained performance gains, essential for pedagogical applications. Moreover, uncontrolled confounders may have influenced the results. For instance, participants may have improved performance due to the Hawthorne effect-motivation from being observed and using novel technology. Similarly, instructors' enthusiasm for the digital intervention may have enhanced the experimental group's engagement. As these factors were uncontrolled, some gains may reflect these biases rather than the technology's effects. Future studies should control or measure these factors (e.g., by blinding intervention aspects or ensuring comparable instructor engagement in the control group) to better isolate the digital teaching model's effects.

## 5 Conclusion

This study demonstrates that a digital table tennis classroom teaching model can significantly enhance university students' physical health and athletic performance. Participants using digital tools and ball-serving robots showed greater improvements in speed and endurance than the traditional instruction group. Notably, the experimental group's 50-m sprint times decreased significantly, and their endurance run pass rates rose by 13.26%, outperforming conventional methods. These results underscore the potential of integrating advanced digital technologies into sports training to enrich physical education and boost student fitness outcomes.

Given these promising results, a key recommendation is to incorporate technology-based digital sports training into national curriculum frameworks. Embedding digital training programs into physical education curricula will ensure consistent adoption and sufficient resource allocation for schools nationwide. This policy-level integration would not only standardize best practices but also motivate instructors to incorporate interactive multimedia content and intelligent training devices into their daily teaching. Ultimately, aligning digital sports programs with national education standards will expand their adoption and effectiveness, fostering a more technologically adept and athletically capable student population.

Continued research is essential to maintain and build on these findings. A key direction is to conduct longitudinal studies on AI-driven digital sports training models to determine whether initial performance and fitness gains persist over time. For example, future studies should investigate the long-term impact of AI-driven training programs on skill retention and physiological adaptations, expanding the scope of digital sports education research. These studies would yield valuable evidence of digital sports training's sustained effectiveness, enabling educators to refine programs for lasting student benefits. In summary, balancing prompt adoption with thorough long-term evaluation will ensure digital sports education transforms physical education and fosters healthier, more skilled students.

## Data Availability

The original contributions presented in the study are included in the article/Supplementary Material, further inquiries can be directed to the corresponding author.
